# Different Sensitivity of Macrophages to Phospholipidosis Induction by Amphiphilic Cationic Drugs

**DOI:** 10.3390/ijms21218391

**Published:** 2020-11-09

**Authors:** Kristin Öhlinger, Markus Absenger-Novak, Claudia Meindl, Jennifer Ober, Eleonore Fröhlich

**Affiliations:** 1 Center for Medical Research, Medical University of Graz, Stiftingtalstr. 24, 8010 Graz, Austria; Kristin.oehlinger@medunigraz.at (K.Ö.); Markus.absenger@medunigraz.at (M.A.-N.); Claudia.meindl@klinikum-graz.at (C.M.); Jennifer.ober@medunigraz.at (J.O.); 2 Research Center Pharmaceutical Engineering GmbH, Inffeldgasse 13, 8010 Graz, Austria

**Keywords:** inhalation, toxicity, phospholipidosis, macrophages, cationic amphiphilic drugs, high-content screening, inter-species differences, drug screening

## Abstract

Phospholipidosis (PLD), the intracellular accumulation of phospholipids, is an adaptive response to toxic stimuli and serves as an important parameter in the biological assessment of compounds. Cationic amphiphilic drugs are the main inducers of PLD and may impair the function of alveolar macrophages. In vivo and in vitro models are used for PLD screening but the choice of the cellular model may be important because PLD develops in a cell- and species-specific manner. In this study, a panel of different staining (LysoSensor, Acridine Orange, Nile Red, HCS LipidTOX, LysoID) was evaluated in murine (DMBM-2, J774, RAW264.7) and human (THP-1, monocyte-derived macrophages from peripheral blood) cells to identify the most sensitive and easy to analyze staining method and to detect species-specific differences in the reaction pattern. Amiodarone and chloroquine served as inducers of PLD. High content screening was used to compare number, area, and intensity of the staining. Due to the fast staining protocol and the sensitivity of the detection, LysoID proved to be the most suitable dye of the testing. The lower induction of PLD by chloroquine reported in vivo was also seen in this study. THP-1 macrophages, followed by DMBM-2 cells, produced the most similar reaction pattern to human monocyte-derived macrophages.

## 1. Introduction

The alveoles of the deep lung are a target for a variety of possible toxic substances (environmental toxicants or drugs) because of their large surface and the poor protection by mucus of the alveolar epithelium. Toxicants may damage epithelial cells lining the respiratory tract (alveolar epithelial cells) or alveolar macrophages. Up to 10% of patients who receive chemotherapeutic agents develop an adverse drug reaction in their lungs [1]. One of the clinical manifestations is interstitial lung disease (ILD), out of which 2.5–3% of cases are drug induced (DIILD). The morphology of alveolar macrophages in this pathology has been described as “foamy” macrophages. The accumulated material, responsible for the foamy appearance, represents not degraded phospholipids and led to the term phospholipidosis (PLD) [2]. PLD is interpreted as an adaptive cellular response to the toxicant, which may lead to impaired macrophage function. Phospholipogenic compared to non-phospholipogenic compounds induced significantly higher rates of lung alveolitis/pneumonitis [3]. Cellular PLD is thought to be pathognomic because alveolar macrophages of patients with DIILD show PLD. Therefore, the FDA recommends to include PLD screening in the testing of drug compounds and formulation excipients [2]. Cationic amphiphilic drugs (CADs) were identified as the main inducers of PLD and DIILD. These compounds are characterized by one or more hydrophobic rings and a hydrophilic chain containing an amine group that is positively charged at neutral pH. Assessment in rodents is the gold standard but susceptibility to PLD shows not only prominent inter-species but also inter-strain differences. Fischer 344 rats reacted very sensitive to amiodarone, while the sensitivity of Sprague-Dawley and Long-Evans rats was intermediate, and Wistar Han rats reacted not at all with pulmonary PLD [4]. There was, on the other hand, good correlation between reaction of rats in vivo and exposure of alveolar macrophages isolated from this strain in vitro.

Mouse spleen macrophages have been used as a platform to identify PLD-inducing compounds using the fluorescently tagged phospholipid *N*-(7-nitrobenz-2-oxa-1,3 diazol-4-yl)1,2 dihexadecanoyl-sn-glycero-3-phosphoethanolamine (NBD-PE) [5]. This platform was able to identify 26 out of 28 PLD inducing drugs correctly and eight out of eight negative controls. Murine macrophages possess certain advantages for in vitro testing compared to human macrophages. In contrast to their human counterparts, murine and rat macrophages do not need differentiation from monocytes but stay differentiated in culture. Mouse macrophages have a similar phenotype and express similar antigens as human macrophages [6]. They also resemble human macrophages functionally, e.g., regarding phagocytosis of tumor cells [7]. The most common murine cell lines for the study of macrophage function are RAW264.7 and J774 cells. These lines derive from tumor cells and RAW264.7 permanently have an M1 state of polarization [8]. J774A.1 cells are more similar to M0 type and can be differentiated into both polarizations of macrophages [9]. DMBM-2 cells, established from the bone marrow of a C3H/HeJ mouse, are less frequently used macrophage models [10]. 

Despite the common use of murine macrophages for the study of human macrophage reactions, the species-specific sensitivity to PLD in vivo and in vitro suggests that human alveolar macrophages should be the most predictive screening models for PLD in human lung [2,11]. Monocyte-derived macrophages (MDM) obtained from human peripheral blood monocytic cells would be the ideal model because these cells reacted similarly to alveolar macrophages from the same donor [12]. The second best option would be human monocyte cell lines, e.g., THP-1 or U937, which can be differentiated into macrophages by addition of phorbol 12-myristate 13-acetate (PMA) or a mixture of PMA and lipopolysaccharide (LPS) [13]. To avoid overstimulation, which caused a less pronounced reaction to a physiological stimulus, treatment with high PMA concentrations should be avoided [14]. Stimulation with 8 nM (5 ng/mL) PMA was found to be sufficient to induce macrophage differentiation of THP-1 cells and appeared more suitable for the detection of responses to weak stimuli.

Primary human macrophages can be differentiated from peripheral blood mononuclear cells (PBMCs) and stimulated with growth factors to obtain distinct polarizations. Macrophages show a variety of phenotype in vivo but two basic polarizations, pro-inflammatory M1 and anti-inflammatory M2 type are used in vitro. M1 polarization in vitro is obtained by treatment with Granulocyte-macrophage colony stimulating factor (GM-CSF), while M2 macrophages require stimulation with Macrophage-stimulating factor (M-CSF) [15]. Based on the surface marker expression pattern, alveolar macrophages have M1 and interstitial lung macrophages M2 state of polarization [16]. Although fast changes between M1 and M2 type are possible, M1 macrophages are expected to represent better models for alveolar macrophages than M2 macrophages [17].

Amiodarone is not only the most common inducer of DIILD in vivo [18], it also induced, after maprotiline, the highest phospholipid accumulation in U937 cells according to Nile red staining in vitro [19]. Compounds with lower hydrophobicity, like imipramine, chloroquine and fluoxetine, caused lower Nile red staining. Both amiodarone and chloroquine are classical inducers of PLD in vivo and in vitro.

Phospholipid accumulation in lysosomes can be identified by the staining of lysosomes. LysoSensor, Nile Red, Acridine Orange, LysoID, and HCS LipidTOX Green Phospholipidosis detection reagent combined with HCS LipidTOX Deep Red neutral lipid stain are established dyes to assess lysosome size and function. LysoSensor dyes are cell-permeable weak bases that selectively accumulate in acidic vesicles after being protonated. At a low pH they have higher quantum yields, which allows visualization of the lysosomes [20]. Acridine Orange accumulation in lysosomes is linked to the presence of the membrane pump responsible for the maintenance of the low pH [21]. The main application is the detection of autophagosomes [22]. Nile Red binds to both neutral and polar lipids and has been used to differentiate between enzymatically degraded low density lipoprotein (LDL) lipid droplets and oxidized LDL-generated PLD in human macrophages [23]. LysoID dye is sequestered in lysosomes due to specific positively charged titratable groups. It acts through a mechanism that is distinct from commercially available fluorescently labeled phospholipid analogs (e.g., *N*-(-7-nitrobenz-2-oxa-1,3-diazol-4-yl)-1, 2-dihexadecanoyl-*sn*-glycero-3-phosphoethanolamine) and has already been used to assess the effect of CADs in osteosarcoma cells [24]. HCS LipidTOX dyes were developed to indicate the accumulation of neutral or polar lipids and have been used for the analysis of PLD in macrophages [11]. 

Although various cell types were assessed for PLD in different assays, no comparison of rodent macrophages to human MDM regarding sensitivity to CADs and pattern of reaction has been made so far. High-content screening (HCS) is a suitable method to address this question as a variety of parameters and many cells can be analyzed. In this study we compared the reaction of three murine cells lines (DMBM-2, J774, and RAW264.7), one human cell line (THP-1), and human MDM generated from PBMCs to amiodarone as a strong and chloroquine as a weaker inducer of PLD. Firstly, an easy to perform and to analyze by HCS staining protocol resulting in similar intensities of all unstimulated macrophages was identified. Parameters like intensity, area and number of stained structures were used. Subsequently, it was clarified which macrophages showed the most similar pattern to human MDM and whether differences in the reaction to amiodarone and chloroquine were present.

## 2. Results

### 2.1. Characterization of Macrophages (Cell Area, Lysosome Area, Lysosome Number/Cell, Cytotoxicity to CADs)

**Figure 1:**
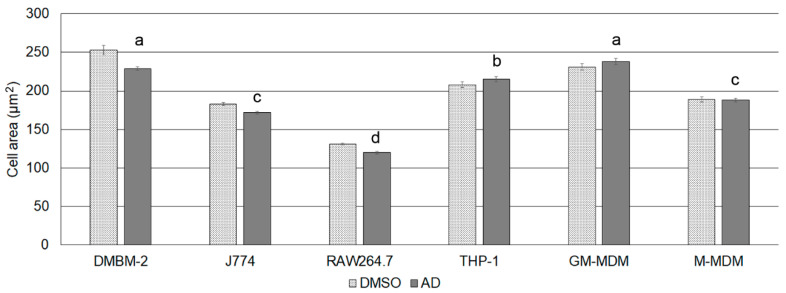
Cell area of macrophages when exposed to the solvent controls. Means ± SEM are shown and groups indicated with letters.

In order to interpret cell-specific differences in the reaction to CADs, cell-specific differences were determined. Cell area was determined in order to correlate the number of lysosomes to cell size and to identify a potential influence of CADs on cell size. Macrophages could be classified into four groups with decreasing size: (1) DMBM-2 and human M1 macrophages, (2) THP-1 macrophages, (3) J774 and human M2 macrophages, and (4) RAW264.7 macrophages (Figure 1). Cell area of medium-exposed cells were identical to the AD controls (data not shown) but significant differences were seen between DMSO and AD exposed cells in the murine macrophages (Table 1).

Lysosome area, staining intensity, and number of lysosomes/cell were determined using LysoID dye. There was no statistical difference between medium exposed and solvent exposed cells regarding the number of lysosomes/cell and lysosome area and between the solvents (data not shown), and differences between the solvent controls were not significant. There was, however, a trend for greater lysosome area in DMSO exposed cells. 

The number of lysosomes/cell decreased in the order DMBM-2 > J774 > RAW264.7, THP-1 > M1 macrophages (Table 1). When normalized to cell area, all murine cells had a density of 0.11–0.13 lysosomes/µm^2^, while human macrophages had a density of 0.05–0.06 lysosomes/µm^2^. The area/lysosome decreased in the order M1 macrophages (0.19–0.22 µm^2^) > THP-1, DMBM-2 (0.12–0.11 µm^2^) > RAW264.7 (0.08 µm^2^) and J774 (0.06 µm^2^). This suggests that, in general, human macrophages have fewer but larger lysosomes/cell than murine macrophages. Furthermore, the extent of the correlation between area and intensity was evaluated. In untreated J774 and THP-1 macrophages, these parameters were closely linked (r = 0.63–0.68), suggesting that larger lysosomes accumulated more dye than smaller lysosomes. Correlation of area and staining intensity in DMBM-2 and RAW264.7 macrophages was much lower (r = 0.42–0.48).

Cell densities after exposure to the different concentrations of the CADs were measured at different positions of the wells. Mean cell densities varied between 60 and 134% of the solvent control and the lowest relative cell densities were recorded for THP-1 and J774 macrophages exposed to 10 µM amiodarone and chloroquine (Supplementary Material, Figure S1). No dose-dependency was seen and variations were high (21–34% of the mean values). This suggests regional variations in cell density rather than dose-dependent cytotoxicity.

To verify the absence of cytotoxicity, conventional cytotoxicity testing using the formation of formazan was used. Viability after exposure to the CADs are listed in Table 2. It can be seen that none of the concentrations caused a significant decrease in viability. Viability of M2 macrophages according to dehydrogenase activity increased at higher concentrations of the CADs. The difference in reaction between M1 and M2 macrophages may be due to reported differences in metabolism [25].

### 2.2. Identification of Optimal Staining 

The optimal staining to reveal inter-cellular differences and inter-compound differences would be a dye that could differentiate sensitively and reliably between untreated cells and CAD-treated cells. Dose-dependency of the signal and similar image acquisition setting for the different macrophages would facilitate inter-cell comparison. In the pilot experiments, one human (THP-1) and one murine (RAW264.7) cell line were evaluated. LysoSensor, Acridine Orange, Neutral Red, HCS LipidTOX Green phospholipidosis detection reagent, and LysoID were assessed in amiodarone- or chloroquine-exposed RAW264.7 and THP-1 macrophages. Staining with LysoSensor differed between solvent-treated and CAD-treated cells but did not show dose-dependency (Supplementary Materials Figure S2). Acridine Orange staining of CAD-exposed cells was different from the solvent but the red/green ratio differed markedly between THP-1 and RAW264.7 macrophages (Supplementary Materials Figure S3). RAW264.7 cells showed a marked decrease, while the reaction of THP-1 cells was poor. The staining pattern of Nile Red and HCS LipidTOX Green Phospholipidosis detection reagent was similar but differences in Nile red staining between CAD-exposed and solvent-treated macrophages were less distinct than with HCS LipidTOX Green Phospholipidosis detection reagent (Supplementary Materials Figure S4). 

**Figure 2:**
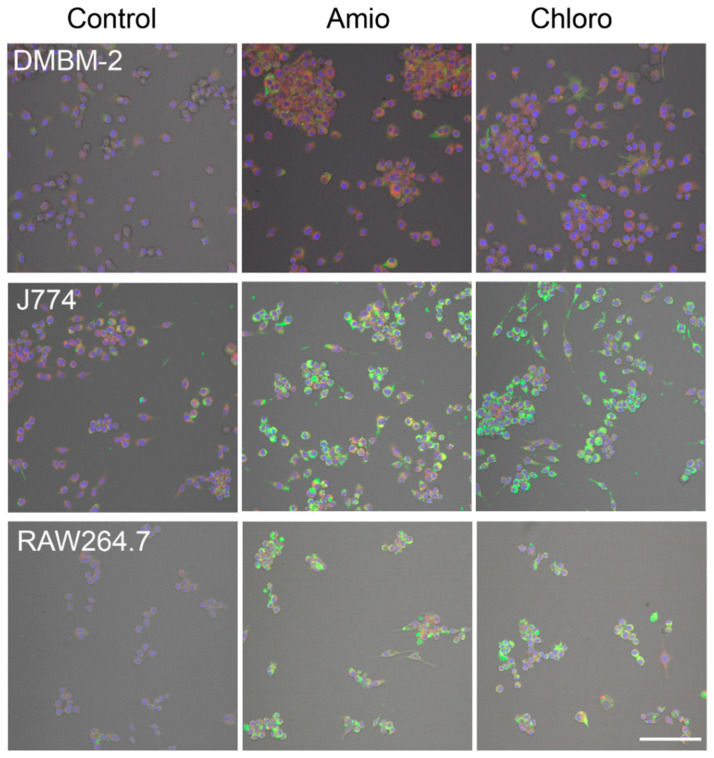
Staining of DMBM-2, J774, and RAW264.7 macrophages with HCS LipidTOX Deep Red neutral lipid stain and HCS LipidTOX Green phospholipidosis detection reagent after exposure to solvent (control), 7.5 µM amiodarone (Amio), and 7.5 µM chloroquine (Chloro). Nuclei are stained in blue. Scale bar 100 µm.

**Figure 3:**
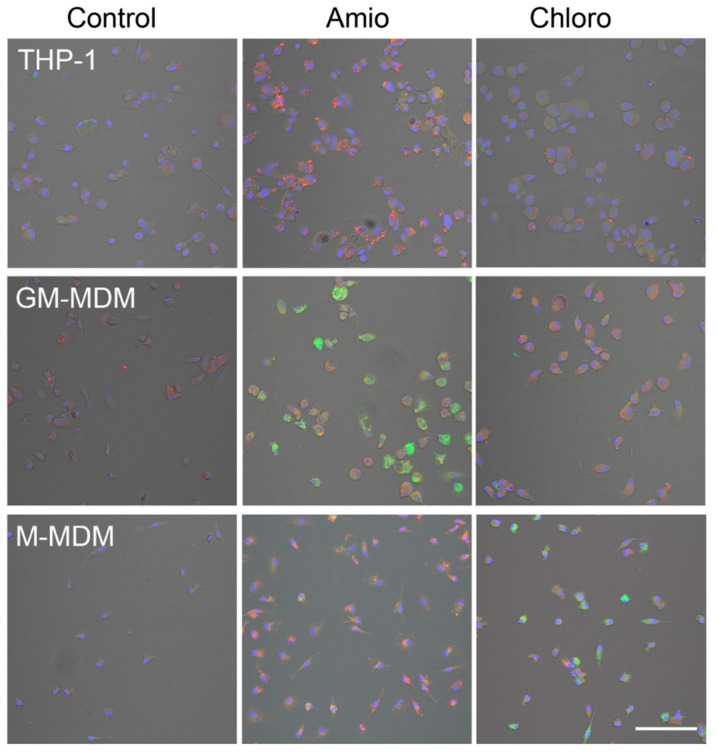
Staining of THP-1, M1 macrophages (GM-MDM), and M2 macrophages (M-MDM) with HCS LipidTOX Deep Red neutral lipid stain and HCS LipidTOX Green phospholipidosis detection reagent after exposure to solvent (control), 7.5 µM amiodarone (Amio), and 7.5 µM chloroquine (Chloro). Nuclei are stained in blue. Scale bar 100 µm.

The basal staining pattern of solvent-treated cells with HCS LipidTOX Green phospholipidosis detection reagent differed between the macrophages but pronounced increase in the staining upon treatment with CADs was seen in murine (Figure 2) and human macrophages (Figure 3). The treatments with both CADs induced only small changes in the staining with HCS LipidTOX Deep Red neutral lipid stain.

**Figure 4:**
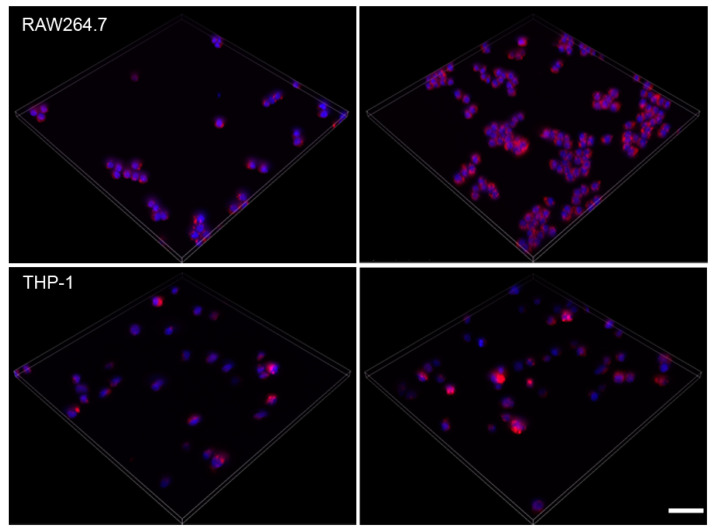
Maximum projection of z-scans from solvent (**left**) and 7.5 µM amiodarone (**right**) exposed RAW264.7 and THP-1 cells stained with LysoID. Scale bar: 50 µm.

The basal staining pattern with LysoID was similar for all macrophages and RAW264.7 and THP-1 are shown (Figure 4). The reaction to chloroquine, in general, was lower than to amiodarone. According to these pilot experiments, HCS LipidTOX Green phospholipidosis detection reagent and LysoID were the most suitable dyes to identify lysosomal changes in a panel of macrophages.

### 2.3. Effect of CADs on Cell Area

Not only lysosomes but also cell size (area) was determined because it has been reported that amiodarone increases the number and size of alveolar macrophages in rats [26]. In our study, both CADs at concentrations ≥2.5 µM increased the cell area of M1 macrophages, at ≥5 µM the area of RAW264.7 cells, and at 10 µM the area of THP-1 cells. The area of M2 macrophages increased upon exposure to ≥2.5 µM chloroquine, while there was no change in cell area upon treatment with amiodarone. No changes in cell area were recorded for DMBM-2 cells and J774. Linear dose-dependency of the increase was only seen for RAW264.7 (data not shown). 

### 2.4. Detection of Phospholipidosis and Neutral Lipids

Since amiodarone did not induce a change in all parameters of the HCS lipidTOX green phospholipidosis detection reagent assay in M1 macrophages, M2 macrophages were also studied with this staining. M2 macrophages were expected to show stronger reactions because it was reported that M2 differentiated U937 macrophages reacted more strongly to amiodarone than M1 macrophages [27]. The reactivity of M2 macrophages to chloroquine was higher than of M1 macrophages but reactivity to amiodarone was lower. M2 macrophages were considerably smaller than M1 macrophages (248 ± 4.0 µm^2^ compared to 188 ± 2.3 µm^2^ consistent with the idea that interstitial lung macrophages are smaller and show M2 polarization [28,29].

**Figure 5:**
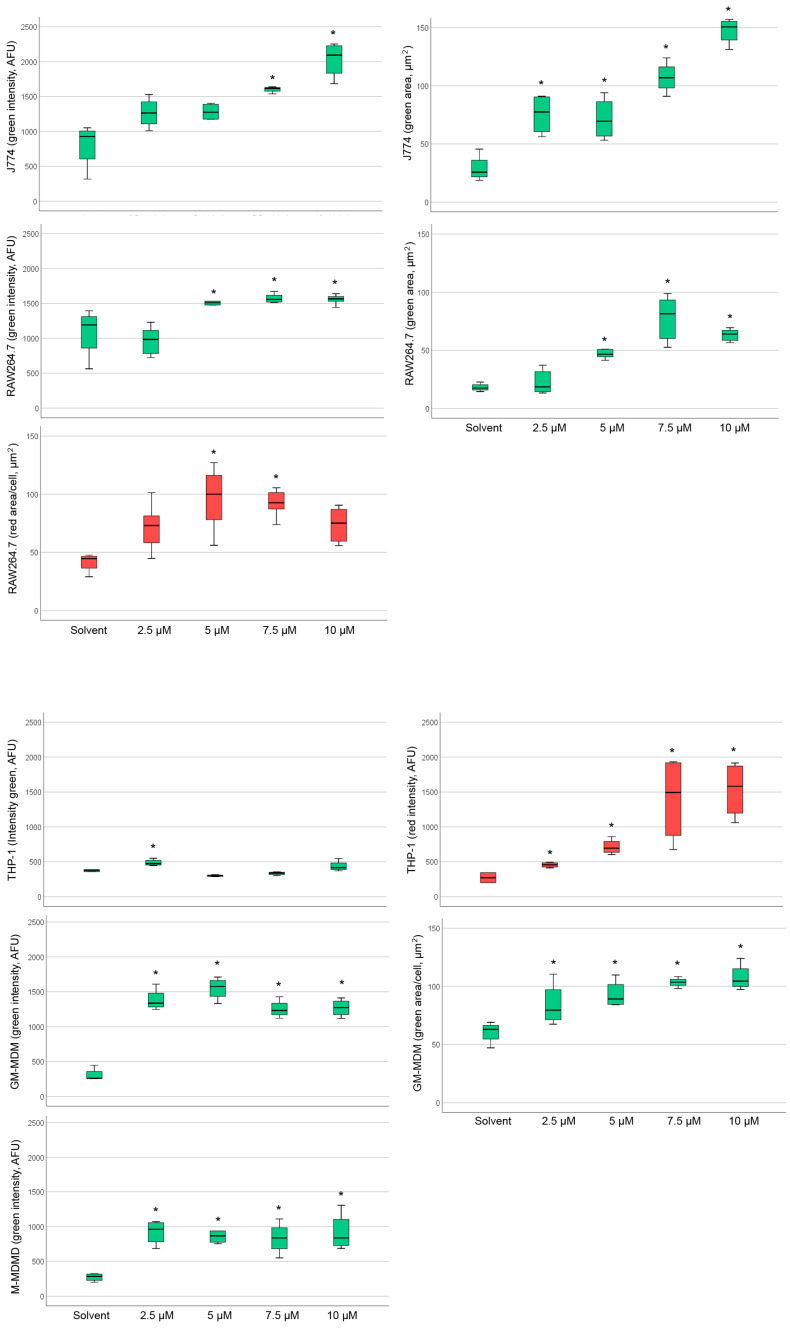
Changes in intensity and area of the green (phospholipid accumulation) and red (neutral lipid accumulation) channel after exposure to amiodarone (Amio) with significant changes indicated by asterisks. Diagrams of murine macrophages are seen in the upper half and human macrophages in the lower half of the Figure. Boxes represent values within the 25–75% quartiles; the whiskers show values outside this range. Abbreviations: GM-MDM: M1 macrophages; M-MDM, M2 macrophages.

**Figure 6:**
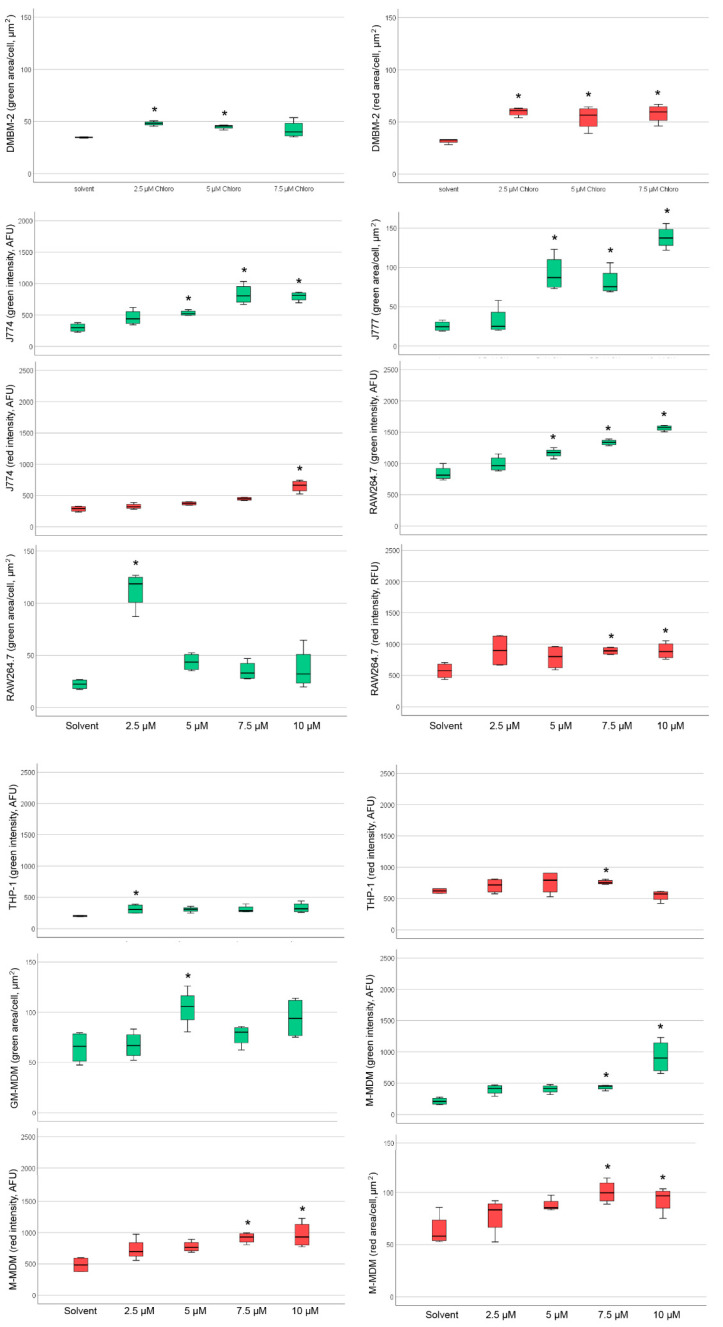
Changes in intensity and area of the green (Phospholipid accumulation) and red (neutral lipid accumulation) channel after exposure to chloroquine (Chloro) with significant changes indicated by asterisks. Diagrams of murine macrophages are seen in the upper half and human macrophages in the lower half of the Figure. Boxes represent values within the 25–75% quartiles; the whiskers show values outside this range. Abbreviations: GM-MDM: M1 macrophages; M-MDM, M2 macrophages.

Analysis of staining with HCS LipidTOX green phospholipidosis detection reagent and HCS LipidTOX deep red neutral lipid staining upon exposure to amiodarone or chloroquine with significant differences to the solvent control can be seen in Figure 5 and Figure 6. 

Table 3 provides an overview of the CAD concentrations, at which the significant increases in staining were noted. Amiodarone increased staining intensity with HCS LipidTOX green phospholipidosis detection reagent in all macrophages, except DMBM-2 (Table 3). Human macrophages reacted more sensitively with intensity changes than murine cells. Staining area was increased in J774, RAW264.7, and M1 macrophages. The reaction of THP-1 cells was unique in the way that the accumulation of neutral lipids was more pronounced than phospholipid accumulation, whereas in the other macrophages little increase in the HCS LipidTOX deep red neutral lipid staining was noted in the cells exposed to CADs compared to the solvent controls.

The reaction of HCS LipidTOX green phospholipidosis to chloroquine in murine macrophages was similar to amiodarone. DMBM-2 cells reacted even more sensitively to chloroquine than to amiodarone (Table 3). In human macrophages, however, the indication of PLD after chloroquine exposure was evident at higher concentrations than for amiodarone. No weaker reaction to chloroquine than to amiodarone was seen in murine macrophages; intensity increased in J774, RAW264.7, THP-1 and M2 macrophages and area in DMBM-2, J774, RAW264.7, and M1 macrophages. Chloroquine increased the intensity of HCS LipidTOX deep red neutral lipid staining in J774, RAW264.7, THP-1, and M2 macrophages and area in DMBM-2 and M2 macrophages. Neutral lipid staining intensity was increased mainly at higher (≥7.5 µM) concentrations of chloroquine. The number of green or red stained objects was also determined, but significant changes were linked to the combined increase in intensity and area and, therefore, did not provide additional information. 

**Figure 7:**
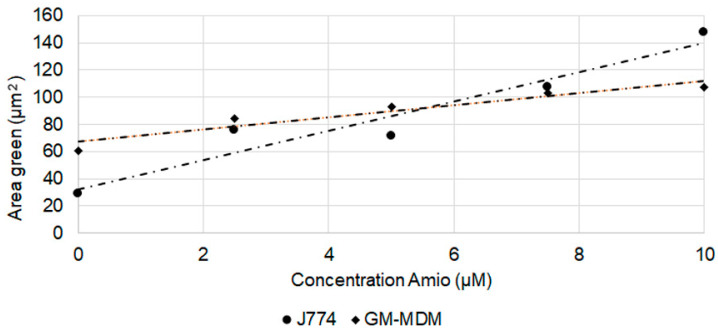
Linear area increase in LipidTOX phospholipidosis detection reagent stained structures upon exposure to amiodarone. Linear relationships with R2 > 0.85 and means ± SEM are shown.

As the reaction of amiodarone shows dose-relationship in vivo [30], dose-dependency of the staining was studied in conditions in which significant differences to the solvent control at concentrations ≥2.5 µM were detected. Linear dose-dependency of HCS LipidTOX green phospholipidosis detection reagent was seen for amiodarone when evaluated by area in J774 and M1 macrophages (Figure 7). For all other cells, significant changes did not show a linear dose dependency or an obvious maximum reaction at a particular concentration. According to the HCS LipidTOX dye staining, both MDMs were more sensitive to amiodarone than the other macrophages (Table 3), while murine cells reacted more sensitively to chloroquine treatment. Another difference was that the murine cells reacted with increases in more parameters to the CADs than human macrophages. 

### 2.5. Effect of Amiodarone and Chloroquine on Number, Area and Staining Intensity of Lysosomes 

Effects of treatment with amiodarone and chloroquine on number, area, staining intensity, and the correlation of area and intensity of lysosomes are summarized in Table 4. Increased staining intensity with LysoID was seen at 2.5 µM in all macrophages. Increase in area was a less robust parameter; only J774 and RAW264.7 cells showed significant increases over the entire concentration range. The lysosome number increased upon amiodarone treatment in all macrophages over the entire concentration range, except for DMBM-2 cells, where no further increase was observed at ≥7.5 µM. 

**Figure 8:**
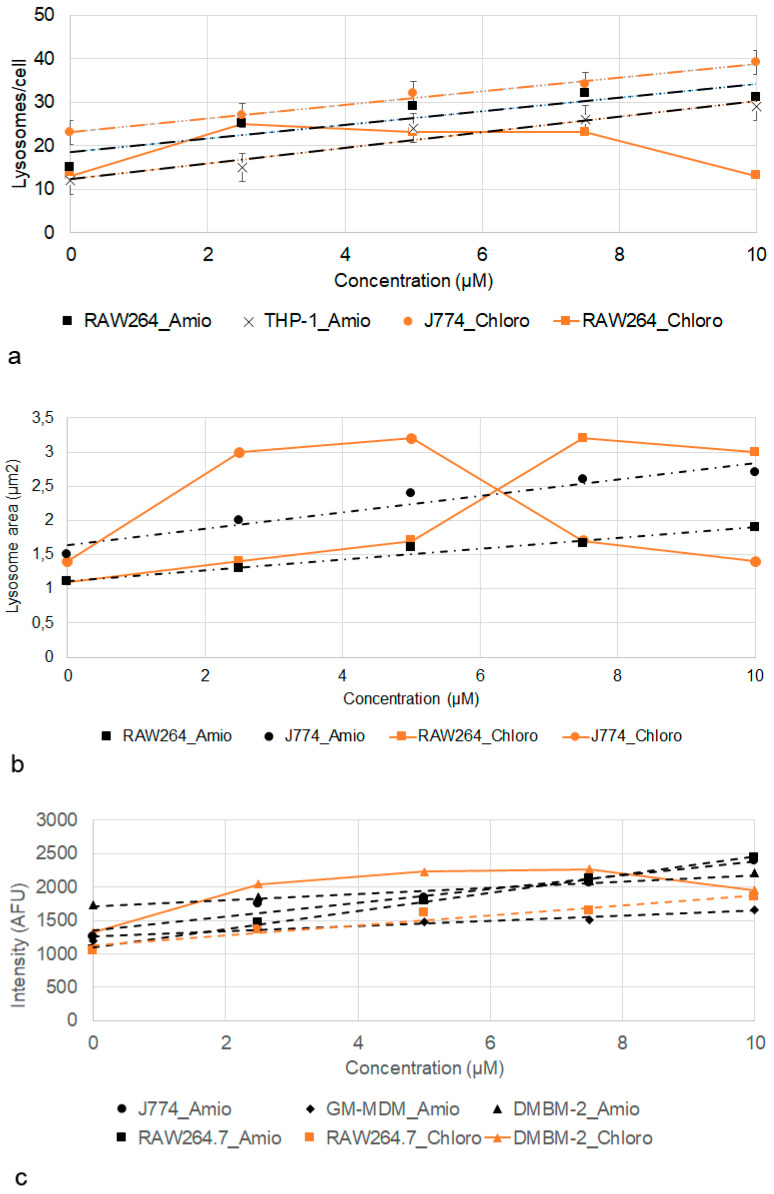
Increase in lysosome number/cell (**a**), area (**b**), and intensity (**c**) according to LysoID staining after exposure to amiodarone (Amio) and chloroquine (Chloro). Linear relationships with R^2^ > 0.85 and means ± SEM are shown.

Linear dose-dependent increase in the response to amiodarone was seen for lysosome number in the case of RAW264.7 and THP-1 (Figure 8a), for area in RAW264.7 and J774 (Figure 8b), and for RAW264.7, J774, M1 macrophages and DMBM-2 for intensity (Figure 8c).

The response to chloroquine was lower in all cells, except RAW264.7 macrophages. A response curve with optimum/plateau was more commonly seen than a linear dose-dependency. A plateau was seen for the number of lysosomes in RAW264.7 cells (Figure 8a), lysosome area in J774 and RAW264.7 cells (Figure 8b), and intensity in DMBM-2 cells (Figure 8c). Conversely, the number of lysosomes in J774 (Figure 8a) and intensity of RAW264.7 cells showed a linear increase (Figure 8c).

Macrophages sensitivity when classified into sensitive (significant increase at ≥2.5 µM), medium (significant increase at ≥5 µM or mixture of significant and non-significant response), and low sensitivity (significant increase at ≥7.5 µM), shows that RAW264.7 and J774 cells were more sensitive than DMBM-2, THP-1 and M1 macrophages to amiodarone. Reactivity to chloroquine overall was lower but the order in sensitivity was the same as for amiodarone.

The correlation of staining intensity to staining area was high (r > 0.5) in J774, M1 macrophages, and THP-1 (Table 5). Solvents increased correlations in the case of DMSO (M1 macrophages) or decreased it for AD (THP-1). This indicates that the solvents did not affect all lysosomes in the same way. Treatment with amiodarone and chloroquine decreased the correlation prominently in J774 macrophages.

## 3. Discussion

### 3.1. Establishment of Macrophage Models and Staining

THP-1 differentiation to macrophages was characterized in this study because protocols with PMA in concentrations of 10–400 nM for durations of 1–3 days have been reported in the literature [13]. Post-culture for 24 h in PMA-free medium is used in most, but not all protocols (e.g., [31]). We used stimulation of THP-1 monocytes for three days with 10 nM PMA with subsequent after-cultivation for one day in the absence of PMA because this concentration was reported as optimal to record reactions to physiological stimuli [14]. Upon the PMA treatment, morphological and functional changes, namely an increase in cytoplasm, in mitochondria, and ribosome numbers, adherence, phagocytosis, accumulation of lipids, resistance to apoptosis, and expression of CD14, CD11b, and CD36 have been reported [32]. Furthermore, THP-1 macrophages showed an increase in CD11c expression from 89.3 to 98.8% [33]. A significant increase in CD14 and CD36 expression in differentiated THP-1 cells compared to THP-1 monocytes was also reported by other authors [34]. Morphological changes, CD expression profile and increased phagocytosis of THP-1 macrophages of this study confirm that the lower concentrations result in good differentiation to macrophages in this study.

Macrophages in this study differed in size and, with the exception of DMBM-2 cells, human macrophages were larger than murine ones, which is consistent with findings by Hoffman et al., who indicated the cell area of human (U937) macrophages with 123–391 µm^2^, and that of rat primary and NR8383 macrophages as 165–265 µm^2^ and 113–210 µm^2^, respectively [11].

In this study, LysoID proved to be the most suitable dye because the staining was fast and settings for image acquisition did not have to be changed because intensity of the staining was similar for all macrophages. Both stainings (HCS LipidTOX Green phospholipidosis detection reagent and LysoID), on the other hand, led to the same conclusion that murine macrophages reacted more sensitively to the tested CADs than human macrophages. Both dyes have been used already for the screening of PLD [11,24]. Increased staining with HCS LipidTOX green phospholipidosis detection reagent after exposure to amiodarone was not seen in DMBM-2 cells, making this assay less sensitive than the LysoID assay. However, images of LysoID staining had to be taken within a relatively short period (max. 15 min), which may present a limitation for performing high-throughput screening. The classification of macrophages according to LysoID staining was easier than with the combination of HCS LipidTOX stains because less parameter had to be taken into account. The combined staining with the HCS LipidTOX dyes provides, on the other hand, more information than the LysoID assay because enlargement of lysosomes could be the cause for which accumulation of neutral and polar lipids and LysoID is not able to differentiate between the two possibilities. Analysis of the staining of the two HCS LipidTOX stains showed that the main contribution to the increase in LysoID stain is the accumulation of phospholipids.

### 3.2. Action of Amiodarone and Chloroquine on Lysosomes

Minimal, not significant, effects on the lysosome area were detected upon treatment with DMSO, which may be due the action of DMSO on membranes. At much higher concentrations of 15% DMSO (compared to 0.1% as solvent), a 15% decrease in structures accumulating Acridine Orange was detected [35]. 

In contrast to the higher reactivity to amiodarone observed in M2 differentiated U937 cells [27], our M2 macrophages showed lower reactivity to amiodarone. Since only 10% and 12% of the U937 macrophages in that study, expressed the M1 and M2 type-specific markers, respectively, polarization may not be the main reason for the higher reactivity to amiodarone. In vivo, PLD was described preferentially in alveolar macrophages located in the intra-alveolar space in humans and other mammals [36,37]. It was further noted that cell area but not lysosome area of M2 macrophages increased upon exposure to chloroquine. This may be explained by induction of M1 state in M2 macrophages by chloroquine as reported by Chen et al. [38]. It has also been seen in this study that M1 macrophages were larger than M2 macrophages.

In a study using HCS LipidTOX Green phospholipidosis detection reagent for the identification of changes in rat and human macrophages after exposure to 0.1, 1, and 10 µM amiodarone, the following results were obtained after 24 h of exposure [11]. Rat primary alveolar macrophages reacted differently from NR8383 and human U937 macrophages and, overall, only a subpopulation of the cells showed changes to amiodarone. Of note, no dose-dependency was seen in the primary cells, while cell lines showed dose-dependent effects. Membrane permeability, cell area, area of lysosomes/cell and number of lysosomes/cells were increased in the alveolar rat macrophages, while U937 macrophages reacted to amiodarone with elevated membrane permeability and elevated cell area only at the highest concentration. Amiodarone increased the lysosome number and area per NR8383 cell only at the highest concentration tested. In contrast to NR8383 and human U937 macrophages, phospholipid accumulation (intensity of staining with HCS LipidTOX green phospholipidosis detection reagent) in the primary rat macrophages was low. The human THP-1 macrophages studied with the same assay in this study showed roughly similar results to the U937 cells, namely increased staining intensity with HCS LipidTOX green phospholipidosis detection reagent and increase in cell area. Accumulation of neutral lipids was seen in THP-1 but has not been reported for U937 macrophages. The M1 macrophages reacted in a slightly different way to the primary rat macrophages reported in the study of Hofmann et al. [11], because in addition to increased lysosome and cell area, increased staining intensity with HCS LipidTOX green phospholipidosis detection reagent was seen. The main difference from the other study was that according to our analysis, the reaction to CADs affected the entire cell population and not only a subpopulation of cells.

Several studies (e.g., [39]) described swelling of lysosomes upon exposure to chloroquine. An increase in area and number were also seen in this study. The finding that the lysosome number increase may also be due to swelling because lysosomes, which were too small to be discerned by fluorescence microscopy, became detectable. Overall, in this study, chloroquine was less active in inducing changes in lysosome morphology and accumulation of phospholipids than amiodarone. The exact mechanism that causes PLD is not known but it is hypothesized that decreased degradation due to inhibition of the phospholipases A1 and A2 and the binding of CADs to phospholipids with the formation of poorly degradable products may play a role [40]. Alternatively, phospholipid synthesis may be increased or transport impaired. The lower effects of chloroquine observed in this study may also be due to the fact that chloroquine was a weaker inhibitor of phospholipase A2 than amiodarone [41]. Another explanation may be the different binding of CADs to phospholipids. In one study dipalmitoylphosphatidylcholine (DPPC) was used and amiodarone bound strongly to the hydrophobic part, chlorphentermine and gentamycin to the hydrophilic part but chloroquine to neither part [42]. Other findings support the hypothesis that amiodarone targets and accumulates in cellular phospholipid bilayer and interferes with the fatty acyl alignment or that some metabolites of amiodarone contribute towards alveolar macrophage toxicity [43]. Finally, it may be possible that a combination of the above-mentioned mechanisms causes the higher sensibility of macrophages to amiodarone.

Most similar to the M1 macrophages reacted THP-1 and DMBM-2 macrophages. THP-1 cells are expected to react most similarly because induction of PLD is species-specific and human and murine macrophages showed distinct differences (e.g., density of lysosomes/cell) in this study. The similarity between the reaction of the bone-marrow derived DMBM-2 cells and M1 macrophages is consistent with data that showed that at the antigen-expression level, human alveolar macrophages are most similar to bone-marrow-derived murine macrophages [6]. The results of this study confirm the species-specificity of the PLD induction and suggest that screening compounds for PLD in murine or rat macrophages in vitro may overestimate their effects in humans.

## 4. Materials and Methods 

### 4.1. Cell Culture

Human THP-1 (CLS) and murine DMBM-2 (DSMZ), J774A.1 (ATCC), and RAW264.7 (LGC Promochem) macrophages were cultured in medium recommended by the supplier at 37 °C, 5% CO_2_ and subcultured at regular intervals. Exposure to amiodarone or chloroquine was performed in the respective culture media (Dulbecco’s Modified Eagle Medium (DMEM) + 10% fetal bovine serum (FBS), 1% penicillin/streptomycin (P/S), and 1% L-glutamine for DMBM-2, J774A.1 and RAW264.7. Roswell Park Memorial Institute (RPMI) 1640 + 10% FBS, 1% L-glutamine, and 1% P/S for THP-1 macrophages RPMI 1640 + 10% FBS, 1% sodium pyruvate, 2 mM L-glutamine, 1% non-essential amino acids (NEAA), 1% P/S for MDM.

Cells were exposed to 0.1% dimethylsulfoxide (DMSO, solvent for amiodarone), 0.1% aqua dest (AD, solvent for chloroquine), and concentrations of 2.5, 5, 7.5, and 10 µM amiodarone hydrochloride (Sigma, Vienna, Austria) or chloroquine diphosphate (Sigma-Aldrich, Vienna, Austria) in the respective media for 24 h.

### 4.2. Differentiation to THP-1 Macrophages

1.0 × 10^6^ THP-1 cells/24 well plate or 2.5 × 10^5^ THP-1 cells/well of an 8-well chamber slide were seeded in RPMI 1640 containing 10% FBS, 1% L-glutamine, and 1% P/S. The well had been coated with 50 µL/well of 10 µg/mL rat collagen type I solution (Sigma-Aldrich) for better attachment. For differentiation, 150 nM phorbol 12-myristate 13-acetate (PMA, Sigma-Aldrich) or 10 nM PMA was added to the media for 72 h, followed by changing the media to a medium without PMA for another 24 h before treatment. 

#### Characterization of THP-1 Macrophages

Undifferentiated and differentiated THP-1 cells of passages 42–52 were characterized using flow cytometry. In pilot experiments, the collection of THP-1 macrophages by cell a scraper was compared to cell harvesting using 0.05% trypsin/EDTA for 10 min at 37 °C. The fraction of viable differentiated THP-1 cells after removal with trypsin was 74.4 ± 2.6% compared to 72.7 ± 6.5% of THP-1 monocytes that did not need detachment. The fraction of single cells was 98.8 ± 0.3% in the macrophage compared to 98.11 ± 0.9% in the monocyte suspensions. The use of cell scraper instead of trypsin treatment decreased the fraction of viable cells by 8% und the fraction of single cells by 0.08% compared to trypsin treatment. The staining with CD36 antibody was reduced by 6.5%. As trypsin appeared to result in better viability and better CD36 staining, cells were collected from the well using trypsin for further experiments. After the addition of RPMI 1640 + 10%FBS, cells were washed once with 1 mL staining buffer containing 3% FBS and 2 mM ETDA. THP-1 monocytes and macrophages were stained in 100 µL staining buffer of a cell suspension containing 500,000 cells with Mouse Anti-Human CD14 PE-Dazzle (Biolegend, dil 1:20), Mouse Anti-Human CD36 FITC (Thermofisher Scientific, Vienna Austria, dil 1:40), Mouse Anti-Human CD11c-APC (BD Biosciences, dil 1:20), and Mouse Anti-Human CD45 APC-Cy7 (BD Biosciences, dil 1:40) for 20 min in the dark at room temperature. Cells were analyzed using CytoFLEX S (Beckman Coulter, Vienna, Austria) with CytExpert software Beckman Coulter, Vienna, Austria).

In order to decide whether treatment with 10 nM was sufficient to induce differentiation to THP-1 macrophages, both exposures were compared regarding their expression of CD markers CD45, CD14, CD36, and CD11c, and their morphology. Overall, differentiation into THP-1 macrophages with PMA resulted in a significant increase in the fraction of CD36+ and of CD14+ cells, while there were no marked changes in CD11c+ cells in THP-1 cells stimulated with 10 nM PMA compared to THP-1 monocytes (Supplementary Materials Figure S5a). Between 99.8 and 100% of THP-1 cells were CD45+ (data not shown). There were no significant differences in the marker expression between use of 10 nM and 150 nM PMA. Furthermore, reaction of THP-1 monocytes in passages 42–52 was similar. Morphological evaluation showed that THP-1 macrophages were adherent and cytoplasmic area increased from 138 ± 1.1 µm^2^ in the monocytes to 210 ± 3.5 µm^2^. THP-1 macrophages at the same microscope setting showed a greater extent of lipid accumulation than THP-1 monocytes (Supplementary Materials Figure S5b). Morphology of the cells was heterogeneous and no obvious differences were discernible between the two differentiation protocols (10 nM PMA and 150 nM PMA). Based on this testing, differentiation of THP-1 with 10 nM PMA was used for all exposures to the CADs.

### 4.3. Generation of Monocyte-Derived Macrophages (MDMs) 

Leukoreduction system (LRS) chambers are a waste product from the production of platelet concentrates and were obtained from the Department of Blood Group Serology and Transfusion Medicine (Medical University of Graz, Graz, Austria). The content of the LRS chamber was mixed with 25 mL Dulbecco’s PBS (DPBS). This mixture was then layered onto prepared 15 mL Ficoll Paque Plus (GE Healthcare Life Sciences, Freiburg, Germany). After centrifugation (400× *g*, 35 min, RT, without brake), the upper layer (plasma and platelets) was removed and the mononuclear cell layer transferred in two new vials. To remove the other cells, this layer was washed two times by filling the vials up to 10–15 mL with DPBS and centrifugation using 400× *g*, 5 min, RT, without brake. The cell pellet was resuspended in an appropriate amount of RPMI 1640 containing 10% FBS, 1% sodium pyruvate, 2 mM L-glutamine, 1% non-essential amino acids (NEAA), 1% P/S to be counted in the TC20 Automated Cell Counter (Bio-Rad, Vienna, Austria). 2.0 × 10^6^ cells/mL cells were seeded in T175 cell culture flasks and incubated for 2–3 h to allow cells to adhere to the plastic surface. 

#### 4.3.1. Differentiation to M1 and M2 State

There are slightly different protocols for differentiation of the MDMs with use of either 5 ng/mL or 50 ng/mL GM-CSF for M1 polarization [44,45] and 10 ng/mL or 100 ng/mL M-CSF for M2 polarization [46,47]. The optimal concentration of CSFs in this study was selected in pilot experiments based on morphology because M1 have mainly round “fried egg” morphology and M2 are spindle-shaped [47]. In the final protocol, after the incubation for 2–3 h (see above), medium was withdrawn and not attached cells removed by washing with PBS. Subsequently, a new medium containing 10 ng/mL GM-CSF (PeproTech Inc., Vienna, Austria) for M1 or 10 ng/mL M-CSF (PeproTech Inc.) for M2 polarization was added for 3 days and replaced with a new medium for another 3 days. After the entire differentiation time of 6 days, the medium was changed to a medium without CSFs and cells were cultured for one more day prior to exposure to the drugs. 

#### 4.3.2. Cytotoxicity Screening Using Measurement of Dehydrogenase Activity 

One hundred µl of medium containing 3.0 × 10^5^ DMBM-2, 4.0 × 10^5^ RAW 264.7, 4.0 × 10^5^ J774, 1.0 × 10^6^ THP-1 or 3.5 × 10^5^ M1 or M2 macrophages/mL were seeded per well of a 96-well plate. After pre-culture for 24 h, the cells were exposed to the CADs, solvents for amiodarone and chloroquine, or 0.5% Triton X-100 as toxic (positive) control. CellTiter 96 AQueous Nonradioactive Cell Proliferation Assay (Promega, Mannheim, Germany) was used according to the manufacturer’s instructions. In brief, 20 μL of the combined MTS/PMS solution was added to 100 μL medium in each well and plates were incubated for 1.5–3 h at 37 °C in the cell incubator. Absorbance was read at 490 nm on a plate reader (SPECTRA MAX plus 384, Molecular Devices LCC, Puch, Graz, Austria). 

In addition to this screening, cell numbers (identified by staining with Hoechst 33,342 staining) in different regions of an 8-well chamber slide were determined and reported as % of solvent control.

#### 4.3.3. Identification of Lysosomes by LysoSensor Staining

After the exposure to samples and controls, the medium was removed and the cells stained with 1 µM LysoSensor™ Green DND-189 (ThermoFisher Scientific) in DMEM + 2% FBS for 5 min at 37 °C. Nuclei were stained by incubation with Hoechst33342 (1 µg/mL) for 15 min at RT. Images were taken immediately with a LSM510 Meta confocal laser scanning microscope (Zeiss, Munich, Germany) with ex 405 nm/Em BP420–480 nm for the blue and ex 488 nm/Em BP 505–550 nm for the green channel. 

#### 4.3.4. Measurement of Membrane Potential by Acridine Orange Staining

After the exposure to samples and controls, cells were washed and loaded with 1 µM Acridine Orange (Sigma-Aldrich) in PBS for 30 min at 37 °C. After a rinse in PBS, images were taken at LSM510 (Zeiss) with ex 485 nm/em 520 nm for the green and ex 584 nm/em 612 nm for the red channel. 

#### 4.3.5. Lipid Accumulation by Nile Red Staining

After the exposure to samples and controls, the cells were stained with 2.5 µM Nile Red (Sigma-Aldrich) in DMEM + L-Glut without phenol red for 3 min at 37 °C. After washing with PBS, cells were viewed by an A1R confocal microscope (Nikon CEE GmbH, Vienna, Austria) at ex 561 nm/em 595 nm.

#### 4.3.6. Neutral Lipid and Phospholipid Accumulation by HCS LipidTOX Dyes

HCS LipidTOX Green Phospholipidosis detection reagent (ThermoScientific, Vienna, Austria; dilution 1:1000) was added to the exposure medium containing either the samples or controls for 24 h. Cells were rinsed in PBS, fixed with 4% buffered paraformaldehyde for 20 min, and incubated with HCS LipidTOX Deep Red neutral lipid stain (ThermoScientific, dilution 1:1000) and Hoechst 33,242 dye (1 µg/mL) for 30 min at RT. Cells were rinsed again in PBS and viewed by A1R confocal laser scanning microscope (Nikon) with the following settings: ex 403 nm/em 450 nm (blue), ex 488 nm/em 525 nm (green), and ex 643 nm/em 700 nm (deep red). Nikon software NIS-Elements software AR 4.60.00 64 bit was used for image analysis. Exposures were performed in duplicates and four regions of interest (containing 2000–6000 cells) were analyzed using NIS Elements software.

### 4.4. Lysosome Morphology by LysoID Staining

After the exposure to samples and controls, ENZO Lyso-ID red detection kit (1:1000; Enzo Life Sciences, Inc., Lausen, Switzerland) together with 1 µg/mL Hoechst 33,342 were added for 20 min at RT. After removal of the staining solution, cells were rinsed once with Kit buffer. After switching to PBS, cells were immediately imaged with a HCS system based on motorized Nikon Ti2 E inverted microscope with C2 confocal system (Nikon CEE GmbH) using Andor Zyla VSC-08691 camera. Hoechst 33,342 was detected at ex 395 nm/em 432 nm and LysoID at ex 555 nm/em 596 nm. Z-stacks were taken at 0.5 µm and 2500 cells were analyzed for number of lysosomes/cell using NIS Elements software with JOBS module. Histograms and significances generated by analysis of 10,000–100,000 DMBM-2 macrophages were compared and no differences regarding significant changes identified. Analysis of 15,000 (THP-1), 30,000 (RAW264.7, J774, MDM), and 40,000 (DMBM-2) cells is presented. For identification of the correlation between intensity and area in the LysoID staining, Spearman’s rank correlation is given. Coefficients between 0.5 and 0.7 were regarded as moderate correlation [48].

### 4.5. Statistics

Data were analyzed using IBM^®^SPSS^®^ Statistics 25 windows 10. Kolmogorov-Smirnov Test was used for a test of normality. For not normally distributed data, a Kruskal-Wallace test was used for group comparisons and Mann-Whitney for comparison of two samples. For normally distributed data, ANOVA with Tukey post-hoc analysis was used. A *p*-value < 0.05 was regarded as statistically significant.

## Figures and Tables

**Table 1:** Characterization of macrophages regarding cell area, number of lysosomes/cell and area of lysosome according to LysoID staining in the different solvent. Means ± SEM are listed and significant differences to AD exposed cells indicated by asterisk. Abbreviation: DMSO, dimethylsulfoxide; AD, distilled water; GM-MDM, M1 macrophages.

Macrophages	Cell Area (DMSO, µm^2^)	Cell Area (AD, µm^2^)	Lysosome Number (DMSO)	Lysosome Number (AD)	Lysosome Area (DMSO, µm^2^)	Lysosome Area (AD, µm^2^)
DMBM-2	253 ± 6 *	229 ± 2.4	30 ± 0.5	30 ± 0.4	3.5 ± 0.014	3.3 ± 0.009
J774A.1	183 ± 1.8 *	172 ± 1.6	24 ± 0.4	23 ± 0.4	1.5 ± 0.008	1.4 ± 0.006
RAW264.7	131 ± 1.6 *	120 ± 1.7	13 ± 0.2	14 ± 0.2	1.1 ± 0.003	1.1 ± 0.004
THP-1	208 ± 3.8	215 ± 3.3	13 ± 0.4	14 ± 0.5	1.5 ± 0.01	1.5 ± 0.01
GM-MDM	241 ± 4.5	238 ± 4.0	11 ± 0.2	10 ± 0.2	2.1 ± 0.01	2.2 ± 0.01

**Table 2:** Viability of macrophages after exposure to amiodarone (Amio) or chloroquine (Chloro) compared to the respective solvent controls dimethylsulfoxide (DMSO) and aqua dest (AD). Abbreviations GM-MDM, M1 macrophages; M-MDM, M2 macrophages.

Treatment	DMBM-2	J774	RAW264.7	THP-1	GM-MDM	M-MDM
DMSO	100 ± 5	100 ± 2	100 ± 3	100 ± 5	100 ± 9	100 ± 2
Amio2.5	98 ± 3	100 ± 1	103 ± 4	86 ± 3	92 ± 7	101 ± 9
Amio5.0	107 ± 3	105 ± 3	111 ± 2	89 ± 3	89 ± 7	123 ± 11
Amio7.5	104 ± 2	108 ± 2	107 ± 3	93 ± 6	97 ± 8	137 ± 2
Amio10.0	90 ± 4	111 ± 2	115 ± 2	105 ± 3	96 ± 9	150 ± 3
AD	100 ± 5	100 ± 1	100 ± 2	100 ± 4	100 ± 5	100 ± 12
Chloro2.5	97 ± 6	99 ± 1	102 ± 2	97 ± 10	97 ± 8	99 ± 9
Chloro5.0	99 ± 3	107 ± 2	104 ± 5	92 ± 5	96 ± 7	95 ± 11
Chloro7.5	99 ± 7	108 ± 3	102 ± 4	91 ± 5	97 ± 7	108 ± 6
Chloro10.0	98 ± 5	111 ± 4	107 ± 4	97 ± 6	99 ± 4	131 ± 5

**Table 3:** Significant changes (*p* < 0.05) in neutral lipid and phospholipid accumulation upon amiodarone and chloroquine treatment according to intensity, area, and object number of HCS LipidTOX staining dyes. Concentrations, at which the significant effect were noted, are indicated in brackets. Abbreviations: GM-MDM, M1 macrophages; M-MDM, M2 macrophages.

	Amiodarone		Chloroquine	
Macrophage	Phospholipid Staining	Neutral Lipid Staining	Phospholipid Staining	Neutral Lipid Staining
DMBM-2	-	-	Area (2.5, 5 µM)	Area (≥2.5 µM)
J774	Intensity (≥7.5 µM) Area (≥2.5 µM)	-	Intensity (≥5 µM) Area (≥5 µM)	Intensity (10 µM)
RAW264.7	Intensity (≥5 µM) Area (≥5 µM)	Area (5, 7.5 µM)	Intensity (≥5 µM) Area (2.5 µM)	Intensity (≥7.5 µM)
THP-1	Intensity (2.5 µM)	Intensity (≥2.5 µM)	Intensity (2.5 µM)	Intensity (7.5 µM)
GM-MDM	Intensity (≥2.5 µM) Area (≥2.5 µM)	-	Area (5 µM)	-
M-MDM	Intensity (≥2.5 µM)	-	Intensity (10 µM)	Intensity (≥7.5 µM) Area (≥7.5 µM)

**Table 4:** Significant changes (*p* < 0.05) in number, area and intensity of LysoID stained structures upon treatment with CADs are indicated. Changes noted at 2.5 µM and ranging until 7.5 or 10 µM are indicated in bold, changes noted only at the highest concentration of 10 µM written in italic. Inconsistent changes in the concentration range of 2.5–10 µM are shown in plain font. Abbreviation: GM-MDM, M1 macrophages.

	Amiodarone			Chloroquine		
Macrophage	Number	Area	Intensity	Number	Area	Intensity
DMBM-2	2.5, 5 µM	**2.5–7.5 µM**	**≥2.5 µM**	*10 µM*	**≥2.5 µM**	**≥2.5 µM**
J774	**≥2.5 µM**	**≥2.5 µM**	**≥2.5 µM**	2.5, 10 µM	**2.5–7.5 µM**	**2.5–7.5 µM**
RAW264.7	**≥2.5 µM**	**≥2.5 µM**	**≥2.5 µM**	**≥2.5 µM**	**≥2.5 µM**	**≥2.5 µM**
THP-1	**≥2.5 µM**	≥5 µM	**≥2.5 µM**	**≥2.5 µM**	*10 µM*	**≥2.5 µM**
GM-MDM	**≥2.5 µM**	2.5, 5, 10 µM	**≥2.5 µM**	*10 µM*	*10 µM*	**≥2.5 µM**

**Table 5:** Correlation of area and intensity of LysoID-stained lysosomes in medium, DMSO for amiodarone (Amio) and distilled water (AD) for chloroquine (Chloro), and CADs. Strong correlations (r = 0.5–0.7) are indicated in bold. Abbreviation: GM-MDM, M1 macrophages.

	Medium	DMSO	AD	Amio	Chloro
DMBM-2	0.42	0.37	0.41	0.35	0.25
RAW264.7	0.48	0.35	0.48	0.47	0.49
J774	**0.63**	**0.63**	**0.63**	0.47	0.44
GM-MDM	**0.56**	**0.63**	**0.53**	**0.57**	**0.55**
THP-1	**0.67**	**0.65**	**0.53**	**0.66**	**0.63**

## Supplementary Materials

Supplementary Materials can be found at https://www.mdpi.com/1422-0067/21/21/8391/s1.

## Abbreviations

Amio	Amiodarone
AD	Distilled water
CAD	Cationic amphiphilic drug
Chloro	Chloroquine
EDTA	Ethylenediaminetetraacetic acid
DIILD	Drug induced interstitial lung disease
DMEM	Dulbecco’s Modified Eagle Medium
DMSO	Dimethylsulfoxide
DPBS	Dulbecco’s PBS
DPPC	Dipalmitoylphosphatidylcholine
FBS	Fetal bovine serum
GM-CSF	Granulocyte-macrophage colony stimulating factor
GM-MDM	M1 macrophages
HCS	High content screening
ILD	Interstitial lung disease
LDL	Low density lipoprotein
LPS	Lipopolysaccharide
LRS	Leukoreduction system
M-CSF	Macrophage colony stimulating factor
M-MDM	M2 macrophages
MDM	Monocyte-derived macrophage
NEAA	non-essential amino acids
PLD	Phospholipidosis
NBD-PE	*N*-(7-nitrobenz-2-oxa-1,3 diazol-4-yl)1,2 dihexadecanoyl-sn-glycero-3-phosphoethanolamine
PBMC	Peripheral blood mononuclear cells
PMA	Phorbol 12-myristate 13-acetate
P/S	Penicillin/Streptomycin
RPMI	Roswell Park Memorial Institute
