# Supplementary material for: Different Sensitivity of Macrophages to Phospholipidosis Induction by Amphiphilic Cationic Drugs
Source: Int J Mol Sci. 2020 Nov 9;21(21):8391. doi: 10.3390/ijms21218391 (PMC7664898; doi:10.3390/ijms21218391)
Supplement: Supplementary file 1 [file ijms-21-08391-s001.pdf]

Supplementary Material

Different sensitivity of macrophages to phospholipidosis induction by amphiphilic cationic drugs

Kristin Öhlinger[1], Markus Absenger-Novak[1], Claudia Meindl[1], Jennifer Ober[1], Eleonore Fröhlich[1,2,*]

[1] Medical University of Graz, Center for Medical Research, Stiftingtalstr. 24, 8010 Graz, Austria; Kristin.oehlinger@medunigraz.at; Markus.absenger@medunigraz.at; Claudia.meindl@klinikum-graz.at; Jennifer.ober@medunigraz.at; Eleonore.froehlich@medunigraz.at

[2] Research Center Pharmaceutical Engineering GmbH, Inffeldgasse 13, Graz, Austria

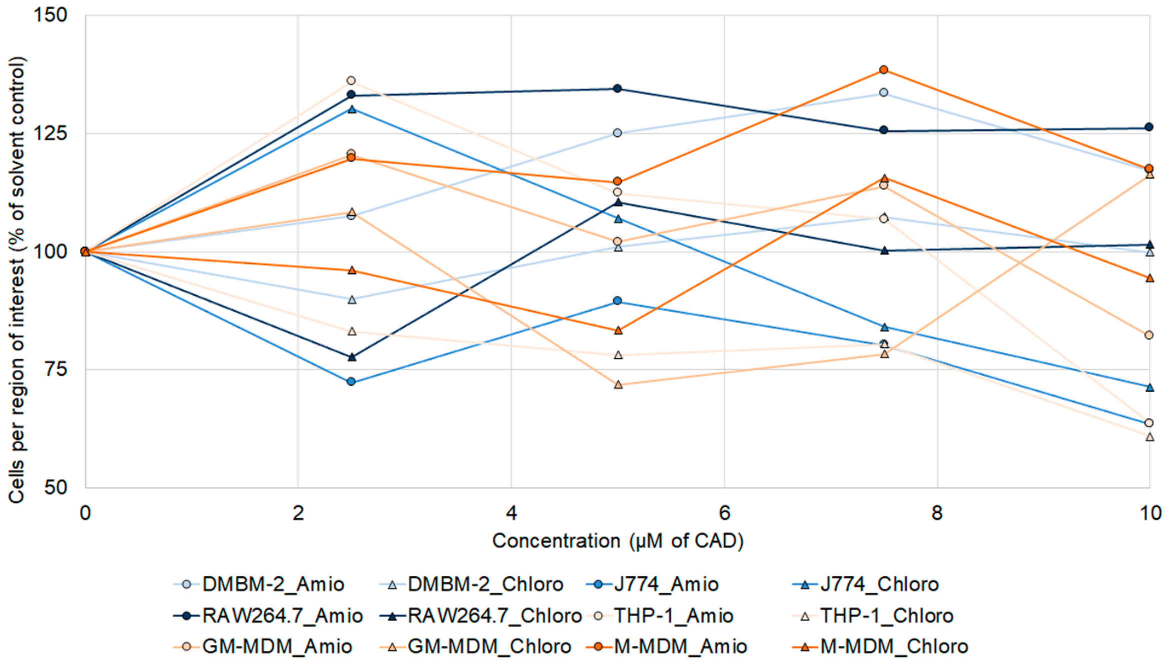

Fig. S1: Dose-dependent changes in cell density. Means (but without indication of standard deviations to increase clarity) of different areas in the culture wells are shown.

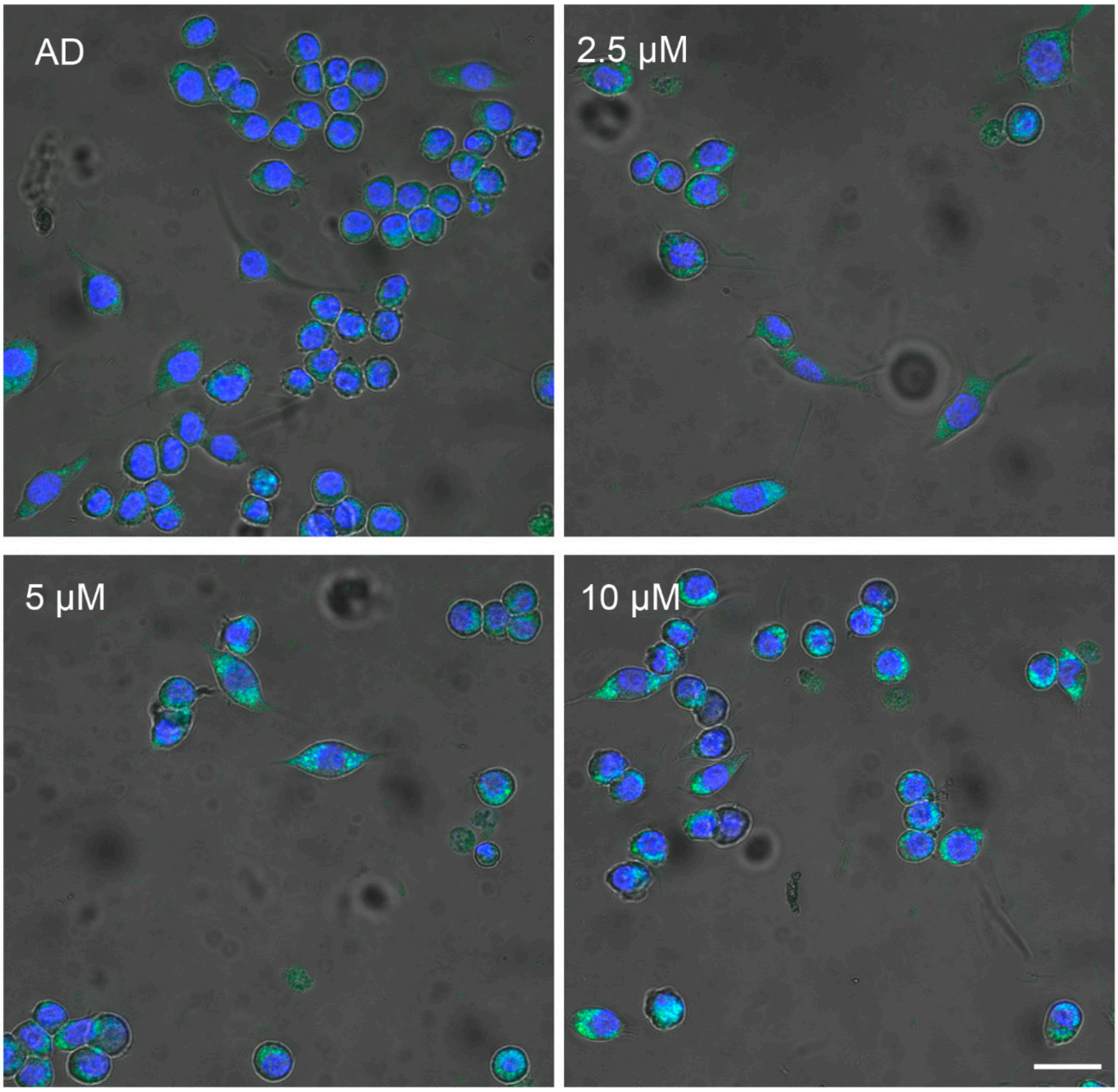

Fig. S2: RAW264.7 cells exposed to solvent control distilled water (AD) and 2.5-10 µM Chloroquine and stained with LysoSensor dye. Scale bar 25 µm.

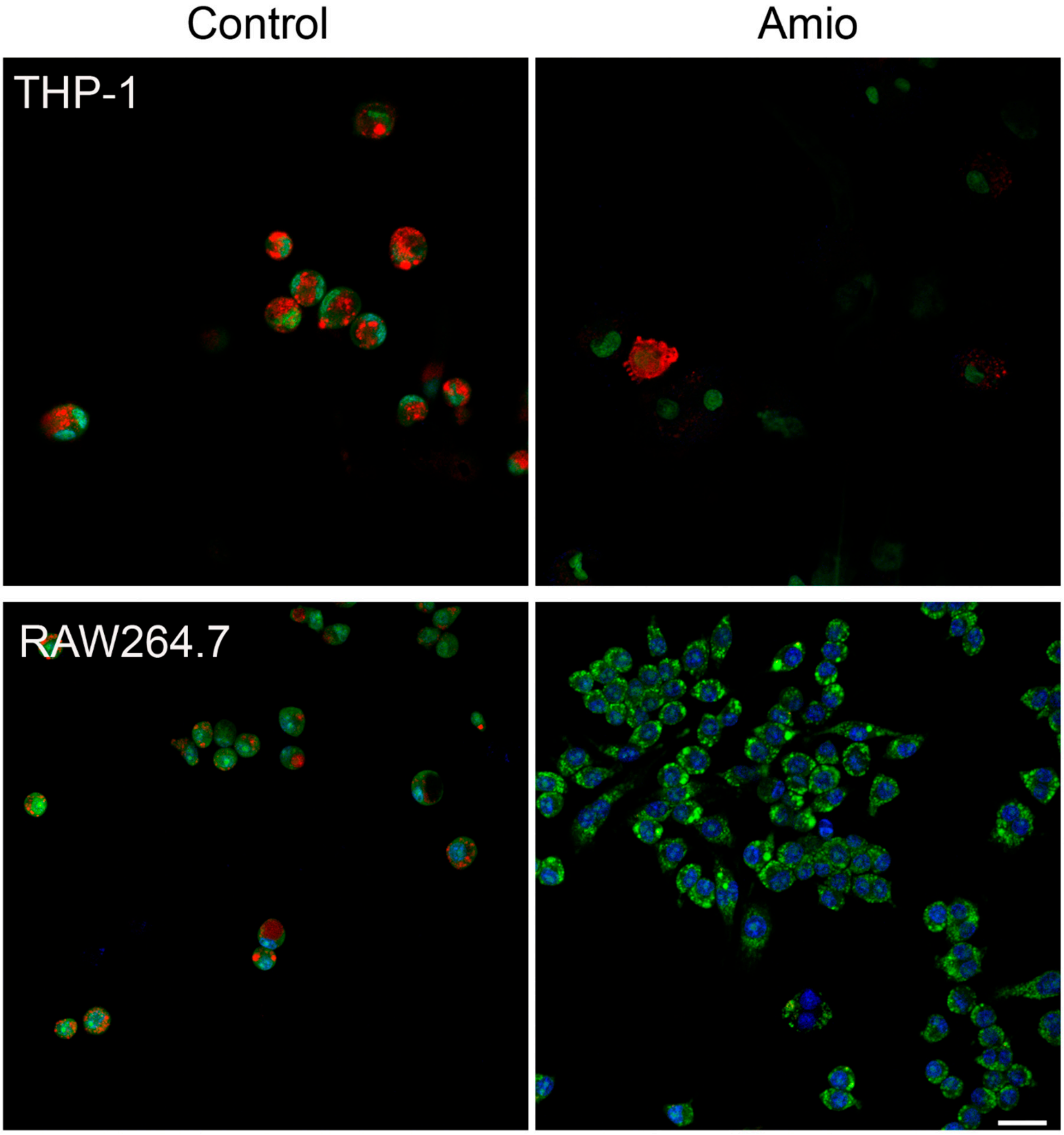

Fig. S3: THP-1 and RAW264.7 macrophages exposed to solvent control DMSO and 7.5 µM amiodarone (Amio) and stained with Acridine Orange dye. Scale bar: 25 µm.

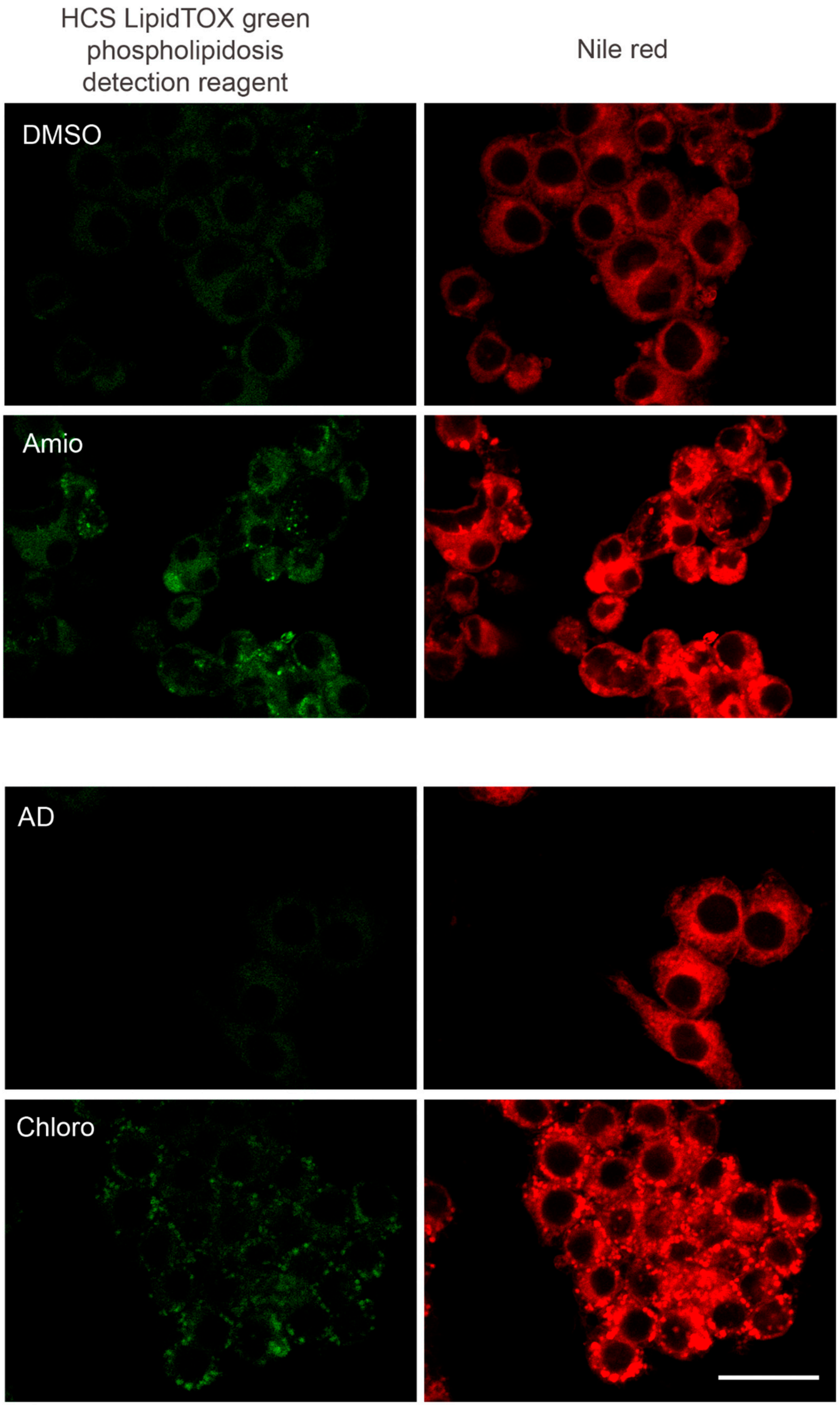

Fig. S4: Co-staining of RAW264.7 cells exposed to solvent (DMSO or AD) and Cads amiodarone (Amio) or chloroquine (Chloro) with HCS green phospholipidosis detection reagent and Nile red. Scale bar: 50 µm.

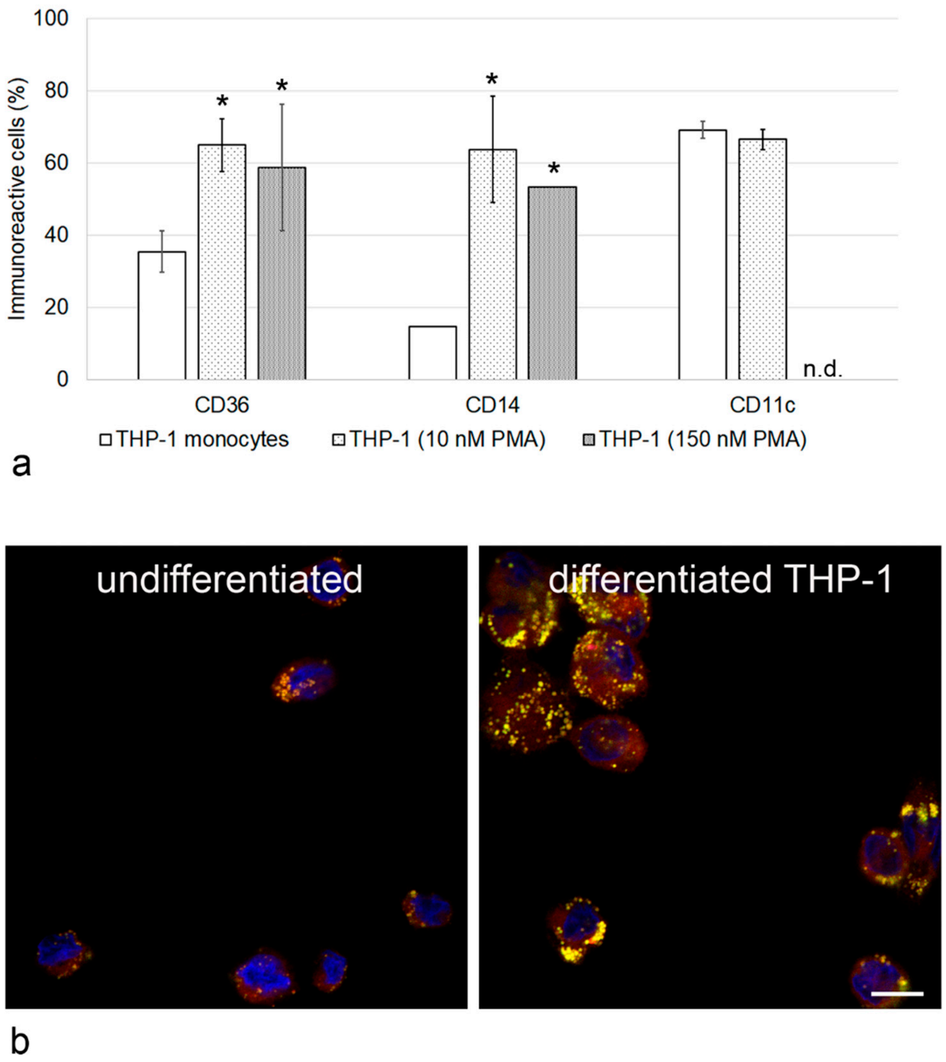

Fig. S5: Changes in CD marker expression of viable cells (a) and of (neutral and phospholipid) lipid accumulation (b) upon differentiation of THP-1 monocytes to macrophages. Scale bar: 20 µm. Abbreviation: n.d., not determined. Differences between THP-1 monocytes and macrophages are indicated by asterisk (n=3).
